# Spatial and temporal features of superordinate semantic processing studied with fMRI and EEG

**DOI:** 10.3389/fnhum.2013.00293

**Published:** 2013-07-01

**Authors:** Michelle E. Costanzo, Joseph J. McArdle, Bruce Swett, Vladimir Nechaev, Stefan Kemeny, Jiang Xu, Allen R. Braun

**Affiliations:** ^1^Language Section, National Institute on Deafness and Other Communication Disorders, National Institutes of HealthBethesda, MD, USA; ^2^Department of Medicine, Uniformed Services University of the Health SciencesBethesda, MD, USA; ^3^Army Research Laboratory, Translational Neuroscience Branch, Human Research and Engineering Directorate, Aberdeen Proving GroundAberdeen, MD, USA; ^4^EOIR Technologies, Inc.Lorton, VA, USA; ^5^PAREXEL Early Phase ClinicalCentreville, VA, USA

**Keywords:** semantic processing, modality independent, superordinate categorization, fMRI, ERP

## Abstract

The relationships between the anatomical representation of semantic knowledge in the human brain and the timing of neurophysiological mechanisms involved in manipulating such information remain unclear. This is the case for superordinate semantic categorization—the extraction of general features shared by broad classes of exemplars (e.g., living vs. non-living semantic categories). We proposed that, because of the abstract nature of this information, input from diverse input modalities (visual or auditory, lexical or non-lexical) should converge and be processed in the same regions of the brain, at similar time scales during superordinate categorization—specifically in a network of heteromodal regions, and late in the course of the categorization process. In order to test this hypothesis, we utilized electroencephalography and event related potentials (EEG/ERP) with functional magnetic resonance imaging (fMRI) to characterize subjects' responses as they made superordinate categorical decisions (living vs. non-living) about objects presented as visual pictures or auditory words. Our results reveal that, consistent with our hypothesis, during the course of superordinate categorization, information provided by these diverse inputs appears to converge in both time and space: fMRI showed that heteromodal areas of the parietal and temporal cortices are active during categorization of both classes of stimuli. The ERP results suggest that superordinate categorization is reflected as a late positive component (LPC) with a parietal distribution and long latencies for both stimulus types. Within the areas and times in which modality independent responses were identified, some differences between living and non-living categories were observed, with a more widespread spatial extent and longer latency responses for categorization of non-living items.

## Introduction

Semantic knowledge includes information about the features, function, and properties of objects and the categories to which they belong (Caramazza et al., 1990; Caramazza and Mahon, 2006). Such information may be extracted from input provided by different sensory systems that can, in turn, either be non-lexical or coded in written or spoken language. In this study, we ask where and when such information converges in the human brain.

Semantic knowledge is hierarchically organized (Mervis and Crisafi, 1982), with superordinate categories having the greatest degree of generality, encapsulating information about more abstract features of a stimulus (e.g., whether it is living or non-living) (Jolicoeur et al., 1984; Rogers and Patterson, 2007). On this basis it might be expected that when subjects are asked to select a superordinate category to which an item belongs, the abstract information derived from different input modalities (e.g., auditory or visual pictures or words) will likely be processed in a unitary, modality independent fashion—in the same brain regions, and at similar times following stimulus presentation. Specifically, superordinate categorical decisions should engage regions downstream from proximal language or visual systems, in heteromodal cortices that process more abstract information, and should occur relatively late in the course of semantic processing. Here we evaluate these hypotheses using two imaging modalities that provide complementary spatial and temporal information. To our knowledge, a single set of experiments evaluating convergent responses in both time and space, while utilizing widely divergent input modalities, has never been conducted.

The anatomical representation of semantic knowledge in the brain has been studied using neuroimaging measures (for a review, see Binder et al., 2009) including both hemodynamic methods such as functional magnetic resonance imaging (fMRI) or positron emission tomography (PET), and electrophysiological methods such as electroencephalography (EEG) and event related potentials (ERP). There has been some scientific debate about the organization, anatomical representation, and timing of these processes, with a principal question being whether there are separate conceptual representations associated with different input modalities (Martin and Chao, 2001) (Paivio, 1971, 1986, 2010) or if information from different modalities converges in a unitary, amodal semantic representation (Caramazza, 1996; Vandenberghe et al., 1996). To address these questions, the hemodynamic methods provide relatively high spatial resolution, ideal for localization of activity in discrete brain regions that may be associated with semantic processing, but less than ideal in providing information about the time course of these processes. On the other hand, EEG/ERP methods allow investigators to precisely characterize the temporal sequences involved in accessing and manipulating conceptual knowledge, but provide only coarse resolution of the spatial origins of this process. In this study, we use both fMRI and EEG/ERP as endpoints in a common experimental paradigm, capitalizing on the advantages of each method to characterize the temporal and spatial features of superordinate semantic processing.

Previous neuroimaging studies have investigated semantic processing at multiple levels and utilized a variety of different input modalities in an attempt to dissociate modality-independent and modality-dependent semantic representations. However, the intended meaning of the term “modality” has not remained constant across studies and the term has been used in a number of ways with several operational definitions (Dilkina and Lambon Ralph, 2012). Most often the term modality has been used to refer to primary sensory (e.g., visual or auditory) inputs, or to lexicality (with stimuli stratified into lexical and non-lexical inputs, e.g., pictures and words). Defined in these ways, a fully balanced experiment would be organized as a matrix containing four stimulus types crossing these two dimensions: non-lexical visual or auditory objects (pictures or environmental sounds), and the written or spoken words that describe them.

Most neuroimaging studies have assessed the impact of input modality have focused investigations within a single dimension, for example differentiating lexical vs. non-lexical stimuli within a single sensory system (e.g., visual objects vs. written words), or using identical lexical items presented in two different sensory modalities (e.g., auditory words vs. visual words). Among the studies using PET and fMRI, those designs that have compared lexical vs. non-lexical stimuli within the visual modality (pictures vs. written words) predominate, and have examined both basic (Sevostianov et al., 2002; Tyler et al., 2003) and superordinate processing (Vandenberghe et al., 1996; Chao et al., 1999; Moore and Price, 1999; Postler et al., 2003; Bright et al., 2004). Other recent work has utilized both auditory and visual input to compare basic level processing of verbal and non-verbal stimuli (Thierry and Price, 2006; Hocking and Price, 2009). Some studies have examined the spatial and temporal features of responses to lexical stimuli in control populations (Marinkovic et al., 2003) and with bilingual (Leonard et al., 2011) and deaf (Leonard et al., 2012) subjects. Others have utilized natural movies to understand the neural basis of object and action categories (Huth et al., 2012).

To date, only a single study (Visser and Lambon Ralph, 2011) used a design that crossed multiple input modalities (comparing spoken words, environmental sounds, and visual pictures) to evaluate superordinate categorization, using fMRI to address questions about spatial location—specifically the contribution of different portions of the temporal lobe in this process. Taken together, however, the findings of hemodynamic imaging studies—particularly in light of the differing definitions of input modality—have been variable. Critically, the issue of where multimodal inputs converge, particularly in the course of superordinate semantic processing, remains largely unresolved.

Several studies have used EEG/ERP methods to address this issue. For example comparing the processing of abstract vs. concrete words, both presented orthographically (Kounios and Holcomb, 1994; Holcomb et al., 1999) and presenting spoken words, pictures and written words (Von Stein et al., 1999). Others have utilized superordinate picture naming paradigms (Schmitt et al., 2000), priming picture naming paradigms (Chauncey et al., 2009) and a recent review highlights one particular component, the N400, as an important marker of semantic memory states (Kutas and Federmeier, 2011). However, to date no study has used a crossed design to evaluate the impact of input modality on semantic processing using electrophysiological methods.

Here we utilize fMRI and EEG/ERP to examine the features of superordinate semantic processing in both time and space, using a cross-modality design that compares responses elicited by visually presented pictures and auditory words. While these conditions constitute only a portion of the matrix outlined above they do not overlap with respect to either sensory input or lexicality and therefore any responses evoked by both classes of stimuli should be true indices of temporal and spatial convergence, independent of input modality, assessed, in this case, by interpreting fMRI and ERP data in tandem.

Superordinate categorization was evaluated as follows: visual pictorial stimuli depicting either living or non-living items [selected from the Snodgrass–Vanderwart picture set (Snodgrass and Vanderwart, 1980)] and auditory lexical stimuli, (spoken names of the Snodgrass–Vanderwart pictures) were presented along with pseudo-stimuli in both visual and auditory modalities. In each trial, subjects were asked to indicate whether stimuli were devoid of meaning (i.e., represented pseudo-stimuli) or, if real, whether the object or word represented a living or non-living item. Pseudo-stimuli were designed to control for lower level sensorimotor processes while minimizing semantic content (see Methods): auditory pseudo-stimuli consisted of words manipulated so that they were recognizable as human voice but lacked any phonological or phonotactic structure that could be matched with real entries in the mental lexicon. Pictures were scrambled so that pseudo-stimuli contained the same spatial frequencies as real items but lacked complex features that could be recognized and matched with stored visual representations of real objects. Both sets of pseudo-stimuli were normed (in a separate population, prior to the imaging studies) to exclude items that sounded or appeared to be real. Thus, in either modality, items should be identified as pseudo-stimuli with little if any further semantic activation, controlling for low-level characteristics of the stimuli. When items are identified as real, subjects extract information with the explicit goal of assigning that item to a superordinate category representing its most abstract semantic features (Mervis and Crisafi, 1982). In both modalities, we identify the regions and times in which these categorical decisions are made and can therefore pinpoint the common responses that are independent of input modality. Pictures and words (along with the respective pseudo-stimulus controls) were presented in separate runs in both EEG/ERP and fMRI experiments. The same paradigm (using distinct but matched sets of stimuli) was run in both experiments. A conjunction approach was used to identify significant, overlapping responses (real vs. pseudo, living vs. non-living) in each.

We predicted that processing more abstract superordinate information should elicit shared responses in higher order, heteromodal areas of the brain and late in course of categorical decision making. Specifically, for fMRI, we hypothesized that responses elicited by superordinate categorization of both pictures and spoken words would be found association areas, specifically the angular, fusiform, and middle temporal gyri and anterior temporal lobe, consistent with previous studies suggesting that these regions are critical for modality-independent semantic processing (Vandenberghe et al., 1996; Brownsett and Wise, 2010; Uddin et al., 2010; Visser and Lambon Ralph, 2011; Cabeza et al., 2012). We hypothesized that ERP components evoked by both auditory words and visual pictures would roughly correspond to the modality-independent temporoparietal responses identified by fMRI and have properties similar to the late positive components (LPC) revealed in previous studies associated with categorical decisions (Mehta et al., 2009) and complex semantic processing (Duzel et al., 1999; Friederici, 2002; Curran and Dien, 2003; Fuggetta et al., 2009; Hajcak et al., 2009; Kissler et al., 2009; Mehta et al., 2009). Thus we predicted modality independent ERP responses would have latencies and morphologies similar to LPCs. Moreover, we predicted that these late ERP responses would bear the same temporal relationship to the button presses indicating the time at which subjects made their categorical decisions, independent of modality. Finally we expected that, when confined to these times and regions, category specific responses (to living and non-living items represented in pictures and words) would retain some unique, albeit subtle features similar to categorical differences previously reported (Martin, 2007).

## Methods

### Subjects

Nineteen male subjects, age 23–39 years participated in the fMRI experiment and 16 subjects (1 female), age 24–34 years participated in the ERP experiment. All subjects were native English speakers without a history of neurological or psychiatric disorders, and were not taking neuroactive medications at the time of their participation in the study. All were right handed, as assessed through the Edinburgh inventory (Oldfield, 1971) (*LQ* = 85 ± 5.5), with normal or corrected vision. Additionally, all subjects were tested prior to the experiments using a combination of standardized test batteries that evaluated language (Boston Naming Test; Kaplan et al., 1983), working memory (Doors and People Test; Baddeley et al., 1994) and general cognitive performance [Repeatable Battery for the Assessment of Neuropsychological Status (RBANS™)] (Randolph et al., 1998). Subjects' individual results were within normal ranges on each test, thus this sample could be considered to be a healthy population without functional impairment. The study was conducted in accordance with the Declaration of Helsinki, and participants granted their informed written consent prior to participation in accordance with the protocol approved by the NIH Institutional Review Board.

### Stimuli

The experiment included presentation of visual and auditory stimulus sets divided into separate runs. The order of these runs was randomized across subjects to avoid order effects in the group analyses. Two hundred and eighty visual and an equal number of auditory stimuli were used in each of the separate runs: 140 real stimuli for both visual and auditory conditions, and complementing these, 140 pseudo-stimuli (visual or auditory stimuli devoid of semantic meaning) for use as baseline. Visual stimuli were selected from black and white line drawings of well-recognized objects from the Snodgrass corpus (Snodgrass and Vanderwart, 1980). Pseudo-objects were created directly from the selected drawings by mixing image elements using graphics painting software; real and pseudo pictures were therefore matched with respect to spatial frequencies thus ensuring equivalence in terms of the low-level characteristics of the stimuli. Auditory stimuli consisted of the spoken words (names of objects from the Snodgrass' stimulus set) read by a professional actor. Auditory pseudo-stimuli were created by dividing the real word stimuli into 3–5 segments using natural boundaries identified in the acoustic waveform and scrambling these into a random order using with Soundforge software (Sony Creative Software). This generated a stimulus set in which items were matched with real words in frequency and amplitude but were devoid of meaning and while recognizable as human voice, were not identified as lexical items. This allowed for isolation of brain regions unique to semantic processing. Both the pseudo pictures and words were normed such that all pseudo-stimuli were not recognized as “real.” Within both the visual and auditory stimulus sets, a group of 70 items represented living objects (e.g., animals and plants), and 70 represented non-living objects (e.g., tools, vehicles, houseware). Images of living and non-living objects were matched with respect to: visual complexity, degree of familiarity and the frequency of use in the speech of the depicted object name (Stewart et al., 1992; Sartori et al., 1993). The auditory subsets of living or non-living objects were matched with respect to duration number of syllables familiarity and frequency of use in speech (Paivio et al., 1968). While auditory words represented names of visual objects from the Snodgrass' stimulus set, the same objects were not used in the auditory and visual condition. Two non-overlapping visual and auditory stimulus sets, matched according to features outlined above were utilized and randomized across subjects.

### Task

**Figure 1:**
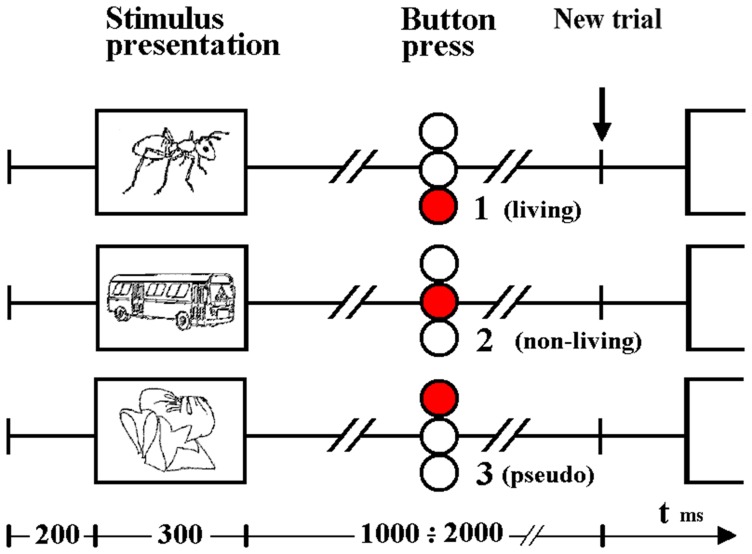
**Timeline illustrating visual stimulus presentation and response.** Subjects indicated a choice between three alternatives (based on whether the item was living, non-living or pseudo) by pressing one of 3 buttons contained in the box held in the right hand. Responses to auditory word stimuli were indicated in the same way although stimulus durations differed (see Methods).

Auditory and visual stimuli were presented in separate runs. In each, 140 real (70 living, 70 non-living) and 140 pseudo-stimulus trials were interleaved. In each trial, subjects were asked to press one of 3 buttons, indicating whether stimuli were (1) devoid of meaning (i.e., represented pseudo-stimuli) and, if not, whether the real object or word represented (2) a living or (3) non-living item. Subjects indicated their decision with a button press using the right hand, and were instructed to do so as soon as possible after stimulus presentation (see Figure 1). Prior to study enrollment, pilot test revealed that subjects performed at an accuracy level consistent with a healthy population (Coppens and Frisinger, 2005). An accuracy was not assessed during the experimental tasks.

Visual stimulus presentation was achieved via projection of the stimulus on a matte screen using a laptop running Presentation® software (Neurobehavioral Systems); subjects viewed the screen through a headcoil-mounted mirror, the visual angle was 7.7° vertically and 10.4° horizontally. All visual stimuli were displayed in the center of the monitor as black contours on a light gray background at a distance of 1.5 m. The duration of each visual stimulus was 300 ms. The verbal stimuli were individually adjusted in loudness so that the participant could clearly hear the sounds. Auditory stimuli were delivered binaurally to the ears by optoelectronic earphones (Resonance Technology, Northridge, CA, USA) and passive noise protection was provided via ear plugs. Auditory stimuli varied from 450 to 900 ms (mean = 670 ms). For both modalities, the interstimulus interval varied from 1.5 to 2.5 s (mean = 2.0 s from stimulus onset).

### Data acquisition

#### Functional magnetic resonance imaging (fMRI)

Blood oxygenation level-dependent contrast (BOLD) functional images were acquired with a 3T whole-body scanner (GE Signa, General Electric, Milwaukee, WI) using a standard quadrature head coil and a gradient-echo EPI sequence. The scan parameters were as follows: *TR* = 2000 ms, *TE* = 30 ms, flip-angle = 90°, 64 × 64 matrix, field of view 220 mm; 22 parallel axial slices were acquired covering the whole brain, with 6 mm thickness. Four initial dummy scans were acquired during the establishment of equilibrium and discarded in the data analysis. Each run comprised 290 volumes, two runs were scanned per subject. The subjects lay supine in the scanner, their heads secured with a padded strap placed across the forehead and secured to the sides of the headcoil, without further mechanical restraint. A button-response box was placed under the subjects' right hand, in a comfortable position with the fingers directly touching the corresponding buttons.

#### Event related potentials (ERP)

Participants were seated in an electrostatically shielded chamber, facing a 34 cm LCD monitor. Visual stimuli were presented to the participant at a 5 visual angle from a distance of 1.5 m. Auditory stimuli were presented at 90 db through a single speaker, located 1.5 m in front of the participant. All electrophysiological signals were recorded using 9 mm sintered silver silver-chloride electrodes. EEG was recorded with a 60-channel electrode cap, conforming to the extended 10–20 electrode placement system and referenced to the nose. Data were continuously recorded using two 32-channel Synamp bioamplifiers, with 0.15–100 Hz bandpass filtering and sampled at 500 Hz. Electrical impedance between the ground and all electrodes was maintained below 5 KΩ. Bipolar leads were placed above and lateral to the left eye, in order to measure the electrooculogram (EOG).

### Data processing

#### Functional magnetic resonance imaging (fMRI)

Preprocessing and statistical analysis of the MRI data were performed using Analysis of Functional NeuroImaging (AFNI) software (Cox, 1996). Functional runs for each subject were motion corrected by realignment using the fourth volume in each run as a reference. Functional images were smoothed with an isotropic Gaussian filter of 8 mm. The data were then normalized by run mean per auditory and visual run. Following preprocessing, a deconvolution analysis using AFNI was performed in order to estimate the hemodynamic response corresponding to each subject for each condition. An ideal waveform time series file was created for each stimulus condition listing the precise timing of each stimulus type. These time series files were then convolved with a gamma function such that an idealized Hemodynamic Response Function (HRF) was determined for each stimulus condition. Regressors included motion parameters (the 6 motion parameters obtained from volume registration), baseline, linear drift and the HRF from the stimulus condition. For statistical comparisons across subjects, the data were standardized into Talairach space in order to account for variability in brain shape and size.

The deconvolution analyses for each subject were submitted to a Three-Way ANOVA, 2 (modality) × 3 (condition) × 19 (subject), over the whole-brain to generate a random effects group analysis. Contrasts of interest included auditory real (living and non-living) vs. pseudo, visual real (living and non-living) vs. pseudo, auditory living vs. pseudo, auditory non-living vs. pseudo, auditory living vs. pseudo, auditory non-living vs. pseudo, visual living vs. pseudo, visual non-living vs. pseudo. Monte Carlo simulations were run using AFNI's AlphaSim program in order to determine the number of contiguous voxels needed to be active in order to achieve a corrected significance level of *p* < 0.01. Whole-brain conjunctions were performed to identify similar voxels activated by real visual images and real auditory words relative to the respective pseudo conditions (*p* < 0.01, corrected). Categorical (living vs. non-living) responses were evaluated using the same conjunction approach (*p* < 0.05, uncorrected). Results were displayed using MRIcron (Rorden and Brett, 2000).

#### Event related potentials (ERP) waveform components

Individual EEG trials were visually inspected and those that contained artifacts or exceeded 100 μ V of EOG were excluded from the analysis. EEG trials were time-locked to stimulus onset and averaged separately for each condition. Waveform peak amplitudes and latencies were derived from a 1400 ms ERP with a 200 ms baseline interval. Latency interval, from which the ERP components peak amplitudes were derived were based on visual inspection of the grand average ERP waveforms associated with living, non-living, and pseudo stimuli, presented in the visual and auditory modalities and are consistent with previously reported LPC component latencies (Kiefer, 2005; Van Strien et al., 2007; Daltrozzo et al., 2012; Kos et al., 2012). An auditory LPC was defined as the maximum positive amplitude between 550 and 900 ms over the left, right, and central parietal region (P1, P2, Pz) (Olichney et al., 2000; Kayser et al., 2003; Van Strien et al., 2007). A visual LPC was defined as the maximum positive amplitude between 400 and 800 ms over the left, right, and central parietal region (P1, P2, Pz) (Kiefer, 2001; Danker et al., 2008).

For both auditory and visual components, separate Two-Way repeated measures ANOVA were conducted in order to determine the effects of stimulus type (real or pseudo) and recording site (left, right, or central) on the amplitudes and latencies of the auditory and visual LPC [consistent with evidence that the morphological differences in visual and auditory waveforms may make separate analyses of ERP latency and amplitude preferable (Holcomb and Neville, 1990)]. For both auditory and visual components, separate Two-Way repeated measures ANOVA were conducted in order to determine the effects of stimulus type (real or pseudo) and recording site (left, right, or central) on the amplitudes and latencies of the auditory and visual LPC. LPCs selectively elicited by living and non-living items were evaluated with separate Two-Way repeated measures ANOVA in order to determine the effects of condition (living, non-living) and recording site (left, right, or central) on the amplitudes and latencies.

Due to potential covariation between experimental conditions, introduced by the repeated measures design, the Huynh and Feldt Epsilon correction was applied to each calculated *F*-statistic. All tests were held to a family-wise error rate of *p* < 0.05. Hypotheses-specific mean comparisons were performed using paired *t*-tests, with Bonferroni corrections in order to maintain the specified experiment-wise type I error rate.

## Results

### Reaction times

**Figure 2:**
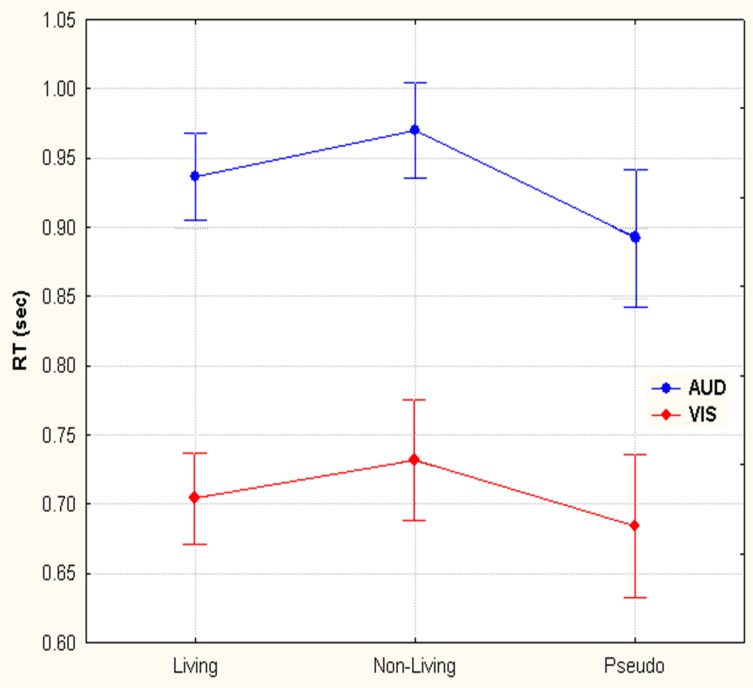
**Reaction time differences between responses to living, non-living and pseudo items for visual images and auditory words.** Selection of visual stimuli was faster than auditory. Within both the visual and auditory modalities, pseudo stimuli were detected first, followed by living and then non-living items.

Two-Way repeated measures ANOVA with modality (auditory—visual) and category (living— non-living—pseudo) as factors demonstrated both main effects, leaving interaction as non-significant. The reaction times from the ERP for auditory words and visual objects demonstrated two principal features: first, button press responses to auditory lexical stimuli were significantly prolonged (933 ms ± 65) (mean ± SD) when compared to visual pictoral stimuli (707 ms ± 72) *F*_(1, 14)_ = 288.1, *p* < 0.001; second, pseudo-stimuli were recognized faster in either modality (893 ± 92 and 684 ± 96 for auditory and visual stimuli, respectively) than living items (936 ± 58 and 704 ± 61), which were themselves recognized faster than non-living (970 ± 63 and 731 ± 80) words or objects *F*_(2, 28)_ = 17.3, *p* < 0.001 (Epsilon = 0.88). (see Figure 2). *Post hoc* Newman-Keuls test revealed, that all 3 categories give significantly different RTs.

### Functional magnetic resonance imaging (fMRI)

#### Convergent responses

**Figure 3:**
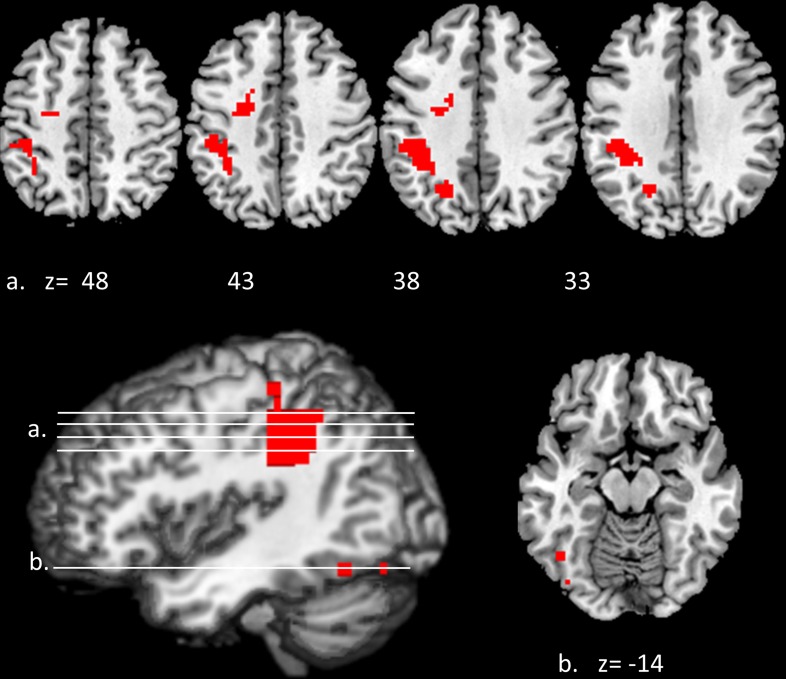
**Modality-independent responses detected with fMRI.** Conjunctions—responses that are elicited by both auditory words and visual pictures—are illustrated. Red indicates voxels that were significant (real—pseudo; *p* < 0.01, corrected) for both modalities. Location of the planes illustrated in the montage are depicted on the surface renderings at the lower left; *z*-axis levels are indicated below each slice.

Convergent responses during superordinate categorization of auditory words and visual pictures were identified by performing whole-brain conjunction analyses utilizing AFNI software as outlined in the methods section. Differences for real visual objects and real auditory words and pseudo-items should reflect the processes by which real items are assessed and assigned to superordinate categories. Conjunctions—voxels in which responses during categorization of real items were significantly greater than pseudo baseline (*p* < 0.01, corrected) for both visual pictures and auditory words—are considered indices of convergent superordinate processing, independent of input modality [i.e., there were no significant differences in the magnitude of the responses elicited by real auditory words or visual pictures (vs. their respective pseudo baselines) in these regions]. Regions associated with superordinate categorization *per se*—regardless of the category selected—included the left posterior parietal cortex including the inferior parietal lobule (angular and supra marginal gyri), intraparietal sulcus and superior parietal lobule, and the left fusiform and left middle frontal gyri. (see Figure 3, Table 1).

**Table 1:** **Modality-independent responses detected with BOLD fMRI**.

**Hemi.**	**Region**	**Brod. No.**	**Visual *t*-score**	**Auditory *t*-score**	***x***	***y***	***z***
L	Inferior parietal lobule (angular/supramarginal gyri)	39/40	4.05	4.93	−36	−46	36
L	Intraparietal sulcus	7	4.52	4.24	−21	−66	35
L	Interior temporal/fusiform gyri	37	3.57	3.26	−39	−78	−14
L	Middle frontal gyrus	6	4.24	4.04	−25	−9	47

Significant differences between responses to real and pseudo stimuli that were common to both auditory and visual domains are tabulated by region of interest with associated Brodmann numbers (Brod. No.), Talairach coordinates (x, y, z) and hemisphere (hemi.) are derived from the conjunction analysis; t-scores at these coordinates are displayed for individual Auditory and Visual contrasts.

#### Category specific responses

We investigated responses selectively associated with living and non-living items within the regions defined by these conjunctions (i.e., those that were activated for both visual non-lexical and auditory lexical items). Although features categorically associated with living and non-living items might be found outside of this sample space, we were primarily interested in the responses within it—i.e., those associated with superordinate categorization that were independent of sensory modality or lexical form. The resulting pattern indicated an overlap between living and non-living categories in the left superior parietal lobule (*x* = −21, *y* = −63, *z* = 38; Vi *t*-score 4.34, Au *t*-score 3.94), supramarginal/IPL (*x* = −36, *y* = −39, *z* = 34; Vi *t*-score 5.39, Au *t*-score 3.71) and left fusiform gyrus (*x* = −45, *y* = −60, *z* = −13; Vi *t*-score 2.60, Au *t*-score 3.17). In each case, the spatial extent was greater for non-living items: activations outside of the areas of overlap, that were significant for non-living but not living items in both modalities were found in adjacent portions of the inferior parietal lobule (*x* = −37, *y* = −48, *z* = 52; Vi *t*-score 2.83, Au *t*-score 3.42) and left medial fusiform gyrus (*x* = −42, *y* = −63, *z* = −14; Vi *t*-score 2.61, Au *t*-score 2.97).

### Event related potentials (ERP)

#### Late responses to auditory words and visual pictures

Amplitudes and latencies of evoked responses that differentiated real and pseudo items were computed for auditory and visual stimuli independently and compared using Two-Way repeated measures ANOVA as outlined in the Methods section. Differences that were associated with both stimulus types (similar in morphology and location and temporally associated with the reaction times indicated by button press) were considered modality independent.

**Figure 4:**
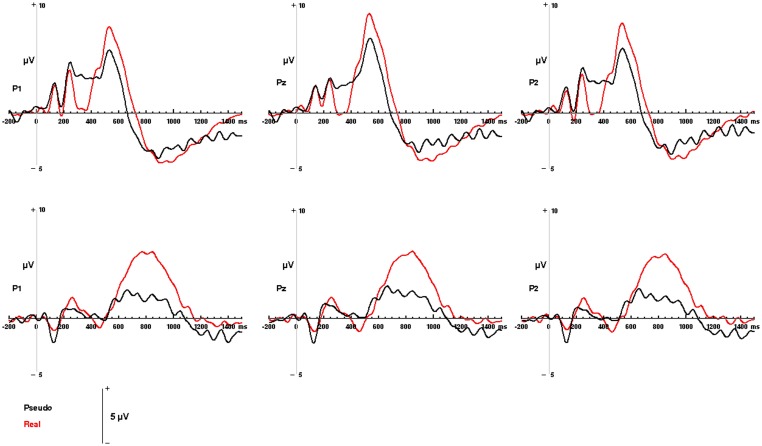
**Modality-independent responses detected with ERP.** Responses to real (living + non-living, red) and pseudo (black) stimuli are illustrated for auditory words (**Top**) and visual pictures (**Bottom**). Waveforms depicting responses to both auditory words and visual pictures are derived from parietal channels P1, Pz, and P2.

**Figure 5:**
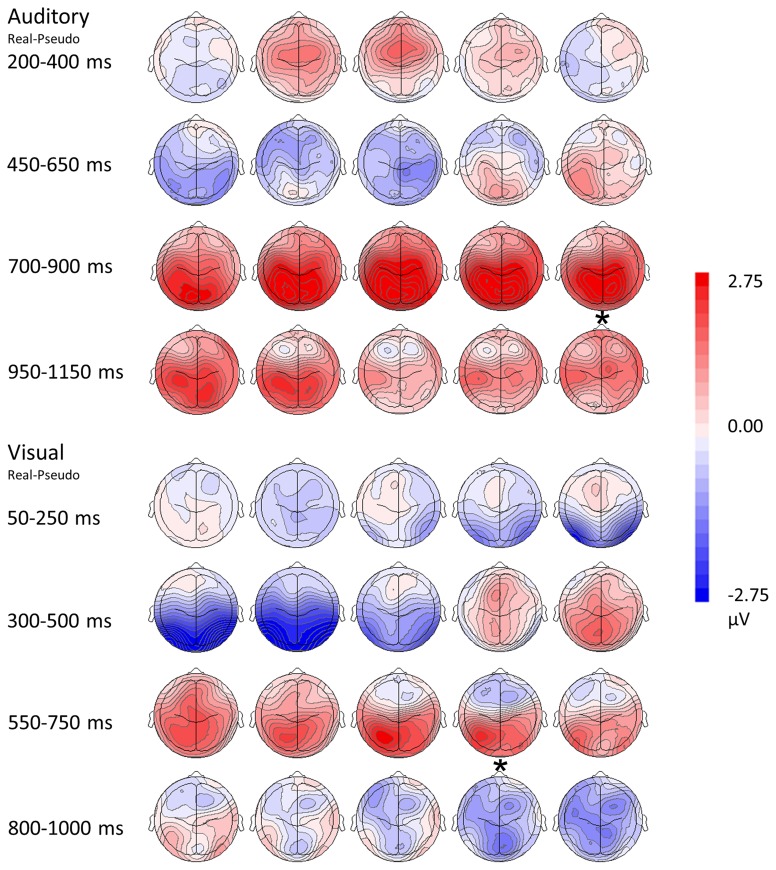
**Topographical rendering of ERP responses over time.** The auditory word condition (real—pseudo) is displayed at the top of the figure, the visual picture condition (real—pseudo) at the bottom. Each image depicts the topographical distribution these of differences as a scalp rendering. These maps are oriented with anterior channels at the top, posterior channels at the bottom; the left hemisphere is represented on the left, the right hemisphere on the right. Responses are sampled every 50 ms. Post-stimulus times are indicated on the left of the figure, with the display adjusted for differences in reaction times (asterisks represents the mean button press times for real items).

Both visual and auditory stimuli, evoked an similar LPC peaking ~150 ms prior to subjects' categorical responses (absolute latencies corresponding to the differences in RT). This robust late positivity was maximal in the left parietal channels. In the auditory domain, the LPC occurred between 550 and 900 ms and over P1, P2, and Pz electrodes (maximal at P1), where real words evoked responses with a significantly greater positive amplitude then pseudo-stimuli *F*_(1, 65)_ = 105.55, *p* < 0.0001 (main effect for condition). No interaction was observed between condition and channel. In the visual domain, the LPC was observed between 400 and 800 ms over P1, P2, and Pz electrodes (maximal at P1). There was a significant main effect for condition with real images evoking responses with greater positive amplitude then pseudo-stimuli *F*_(1, 65)_ = 19.04, *p* < 0.0001. No interaction was observed between condition and channel (see Figures 4, 5). There were no responses selectively evoked by superordinate categorization of real words or pictures other than the LPC.

#### Category specific responses

**Figure 6:**
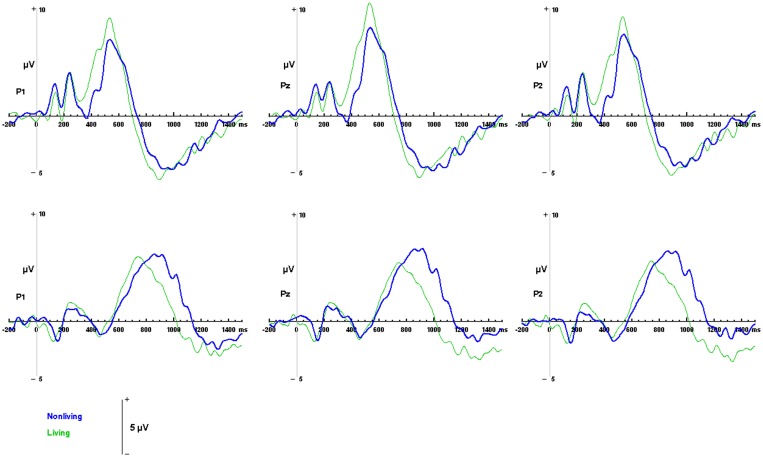
**ERP responses to living and non-living categories.** Waveforms depicting responses to living items (green) and non-living items (blue) are illustrated for auditory words **(top)** and visual pictures **(bottom)** for parietal channels P1, Pz and P2.

Differences between responses to living and non-living items were evaluated within the sample space defined by the LPC (400 to 800 ms over Pz, for visual pictures; 550 to 900 ms over Pz, for auditory words). No significant differences in mean amplitudes were found between components evoked by living and non-living items in either modality (visual: *F*_(1, 13)_ = 2.59, *p* = 0.1318; auditory, *F*_(1, 13)_ = 1.35, *p* = 0.2656 between living and non-living amplitude). However, latency differences between categories were detected. In the auditory domain these were significant, with maximal responses to words associated with living items occurring significantly earlier (756 ms) than those associated with non-living (855 ms) items *F*_(1, 13)_ = 17.61, *p* = 0.001. In the visual domain, responses to pictures of living items (532 ms) also occurred earlier than pictures of non-living items (569 ms), although this difference did not reach statistical significance (see Figure 6).

## Discussion

Superordinate semantic processing requires access to the most abstract conceptual information (Mervis and Crisafi, 1982), making it possible to categorize stimuli based upon general features shared by broad classes of exemplars. The same categorical information may be derived from multiple input modalities (e.g., stimuli that are either visual or auditory, lexical or non-lexical). We proposed that because of the abstract nature of such information, these inputs are likely to converge and be processed in a common set of heteromodal brain regions at similar time scales, late in the course of superordinate categorization.

The present study tested this hypothesis by utilizing neuroimaging techniques sensitive to temporal and spatial features of brain activity in an experiment designed to minimize the degree of overlap between input modalities and control for low-level characteristics of the stimuli, so that any common responses should precisely identify finite areas and times of convergence. Consistent with our hypothesis, fMRI and ERP results revealed that information from different modalities converges onto a functional network that is activated late, independent of modality or stimulus type, consistent with the establishment of a superordinate semantic representation.

In addition, we detected responses that were selectively associated with presentation of visual pictures and auditory words (see Supplementary Material for results). For fMRI these were unsurprisingly found in regions that play a role in visual object recognition (the inferior occipital, and fusiform gyri) (Borowsky et al., 2007) or auditory-lexical processing (left superior and middle temporal gyri) (Hickok and Poeppel, 2007), respectively. For EEG/ERP, these responses occurred earlier in each trial and were located in roughly same distribution (occipital and left temporal channels) as the fMRI responses. In both cases, these early responses occurred ~300 ms prior to the late posterior components that were evoked by both input modalities. It is possible that these responses may reflect basic rather than superordinate semantic processing in regions that are more closely associated with auditory or visual modalities or process lower level lexical or pictorial information (Jolicoeur et al., 1984; Tanaka et al., 1999). However, it is difficult, given the design of the present experiment to disambiguate the relative contributions of sensory or lexical features to these earlier spatially discrete responses. In order to differentiate these, future experiments might additionally include written words and auditory objects as stimuli, making more direct comparisons, and unambiguous interpretations, possible. In any case, this was not our goal here; our paradigm was instead designed to maximize differences between input modalities in order to provide information about the site and timing of their convergence.

### Superordinate categorization

#### Functional magnetic resonance imaging (fMRI)

Regions in which responses to visual pictures and auditory words overlapped were strongly left-lateralized and included left fusiform and portions of the middle frontal gyri; the strongest responses were in the left posterior parietal cortices—angular and supramarginal gyri, intraparietal sulcus, and superior parietal lobule. Collectively the parietal regions are crucial for multisensory integration and association of semantic information, consistent with a role in modality-independent access that supports superordinate categorization. For example, the angular gyrus is a hetermodal region, involved in many aspects of cognition (Uddin et al., 2010; Seghier, 2013) essential to language and semantic processing and their intersection (Vigneau et al., 2006; Binder et al., 2009; Brownsett and Wise, 2010). A recent review indicates that this region acts as a cross-modal hub processing conceptual information that is received through input from multiple sensory modalities (Seghier, 2013). Our results are consistent with these attributes. The supramarginal gyrus and superior parietal lobule are also heteromodal areas that receive input from both auditory and visual unimodal association cortices (Booth et al., 2002), and have been implicated in the neural representation of semantic knowledge (Binder et al., 2009; Chou et al., 2009; Wu et al., 2009). In addition, the superior parietal lobule/inferior parietal sulcus play a role in information synthesis and knowledge recall that may be necessary for high level modality-independent integration and semantic association (Buchel et al., 1998; Bright et al., 2004; Davis et al., 2004; Binder et al., 2009).

We also detected modality independent responses in the fusiform gyrus, which appears to play a key role in conceptual representations that are independent of sensory modality, and are activated by both pictures and words (Caramazza and Mahon, 2006; Martin, 2007; Binder et al., 2009) and in the middle frontal gyrus, a region vital to decision making and choice execution (Heekeren et al., 2006). Thus, these regions are all essential for categorical decisions involving multimodal processing of conceptual information.

The concept of a modality-independent semantic core connected to regions that process modality-dependent semantic features is in general consistent with the “distributed plus hub model” described by Patterson and colleagues (Patterson et al., 2007). However, our modality independent activations did not include the anterior temporal lobe, which is the designated “hub” in that model. Instead, in the present experiment, the posterior parietal cortex appears to act as a central region coordinating superordinate representations. It is possible that we may have under-sampled the ventral portions of the anterior temporal lobe in which these authors (Visser and Lambon Ralph, 2011) report conjunctions in a categorization task similar to our own. Interestingly, evidence in favor of the anterolateral temporal cortex as a possible hub for semantic processing is based primarily on neuropsychological data from semantic dementia patients who show selective deterioration of this region and subsequently show decline in basic level categorization (Patterson et al., 2007). But importantly, these patients show less vulnerability when asked to perform superordinate categorical tasks retaining general and typical semantic knowledge. Thus, our results are consistent with these clinical findings, supporting the notion that superordinate categorization may at least in part take place elsewhere, specifically in the posterior parietal cortex.

A recent study examining semantic aphasia suggests the importance of these posterior regions in modality independent access to semantic control (Gardner et al., 2012). Collectively these regions may be part of an extended semantic network since modality independent representations have been reported in the supramarginal gyrus, angular gyrus and posterior superior temporal gyrus area, contributing to semantic connections in the middle temporal gyrus (Booth et al., 2003). It should be noted, however, that the conjunctions we detected in the parietal areas did not extend ventrally into the posterior temporal regions. Thus, although the latter areas are considered crucial for processing lexical semantic information, our results suggest that they may not represent a site of convergence for superordinate information derived from multiple input modalities.

#### Event related potentials (ERP)

The ERP results provide information about the temporal features of semantic processing, revealing a LPC with a parietal distribution for both modalities: that is, the LPC is essentially the same in amplitude and distribution whether the stimuli are visual objects or auditory words, suggesting modality independent responses. Importantly these responses were strongest in channel P1, roughly corresponding to the posterior parietal areas in which convergent activations were detected in the fMRI portion of the experiment. Such a parietally distributed positivity underlying higher-order semantic processing such as categorical judgment has been previously reported (Mehta et al., 2009) and other studies have demonstrated a similar late response with a left posterior parietal distribution that reflects manipulation of complex, task relevant semantic information (Duzel et al., 1999; Von Stein et al., 1999; Friederici, 2002; Curran and Dien, 2003; Fuggetta et al., 2009; Hajcak et al., 2009; Kissler et al., 2009; Mehta et al., 2009). Taken together, the fMRI and ERP results suggest that the posterior parietal cortex may be instrumental in the later, convergent processes that play a role in superordinate categorization.

Since basic level semantic information appears to have a privileged position in the semantic hierarchy, i.e., basic semantic features are processed first (Markmen, 1991; Tanaka et al., 1999), it is possible that the components unique to visual pictures or auditory words (discussed in the previous section) may reflect these processes (although see the caveats outlined in that section). Nevertheless, the hallmark of the LPC is that it occurs late and is evoked by both classes of stimuli, consistent with it being a marker for the convergence and manipulation of more abstract semantic information. Our results indicate that the auditory LPC occurs later (between 550 and 900 ms) than the visual LPC (which occurs between 400 and 800 ms). The latency differences between visual and auditory modalities are consistent with notion that pictures may have more rapid access to semantic networks while the auditory words must first be processed through the mental lexicon (Caramazza and Hillis, 1990; Amrhein et al., 2002; Sevostianov et al., 2002). In addition, physical features of the stimuli may contribute to the longer latencies associated with auditory word stimuli (mean duration 670 ms) compared to the visual pictures (duration 300 ms.) Crucially, however, in both visual and auditory modalities, the LPC precedes the button press response (which reflects execution of the subject's superordinate categorical decision) by the same period of time ~150 ms. These consistent temporal relationships suggest that if the parietal LPC reflects the processing abstract information required to assign stimuli to superordinate semantic categories, that this processing is completed about 150 ms prior to the motor response indexing the categorical decision.

### Category specific responses

#### Functional magnetic resonance imaging (fMRI)

We also evaluated the responses to living and non-living items independent of sensory modality or lexical form, looking for evidence of cortical representations that may be unique to each category. Confining our search to the regions in which convergent responses were detected in the earlier conjunction analysis, our results suggest that while superordinate categorization recruits a common network, there are only minor differences between the representation of living and non-living categories within it. Specifically, selective responses (essentially a more wide spread extent of activation) were detected for non-living items in the inferior parietal lobule and the medial fusiform gyrus. These results are consistent with previous reports demonstrating that non-living items (e.g., tools) elicit enhanced activity in left inferior parietal cortex, relative to animals or faces (Chao and Martin, 2000), and that recognition of non-living objects is selectively associated with activation of the medial fusiform gyrus (Martin, 2007).

#### Event related potentials (ERP)

While the LPCs identified by ERP were similar in amplitude and morphology for both living and non-living words or pictures, there were systematic, category-specific differences in their latencies. Responses to living items occurred significantly earlier than responses to non-living items when these were presented as auditory words. Such a difference was not found with visual pictures since the peak of the LPC for living items was not significantly earlier than that detected for than non-living items. However, these results are consistent with our own reaction time data which demonstrate that living items are selected earlier than non-living items and with the results of other studies in which living items were identified prior to non-living items in both modalities in a picture-word matching task e.g., (Fuggetta et al., 2009) when familiarity, manipulability and level of identification are controlled for (Filliter et al., 2005).

### Summary

It should be noted that one limitation of the present study is that fMRI and EEG data were obtained separately, in different groups of subjects and the sample was primarily male. Future studies would benefit from simultaneous monitoring of fMRI and EEG and by including more female participants. Nevertheless, these data suggest that superordinate categorization recruits a common anatomical network in which there exists a high degree of overlap during processing of individual superordinate categories. The fMRI results suggest that, consistent with previous reports (Chao and Martin, 2000; Martin, 2007) there are areas in the basal temporal and posterior parietal cortices responses that are significantly modulated by semantic category. The ERP results suggest that while although regional specialization may be limited, there are differences in the speed of processing of discrete superordinate (at least living and non-living) categories.

In summary our results support a model for superordinate semantic processing in which information from multiple input modalities converges and is processed in a network of heteromodal regions that is strongly lateralized to the left hemisphere, late in the course of superordinate categorization. The fMRI data provide spatial information that localizes these responses to parietal, basal temporal and frontal cortices. EEG/ERP data provide temporal information roughly confirming the parietal location of the strongest responses seen with fMRI. Crucially, the electrophysiological data indicate that these responses occur relatively late and have the same temporal relationship, for both auditory words and visual pictures, to the button press reflecting execution of the categorical decision. fMRI results suggest that while the posterior parietal areas appear to be specialized for processing superordinate information, responses are only modestly modulated by the categories themselves, while ERP results suggest that categorical differences in these regions may be more prominently reflected in the speed of processing.

### Conflict of interest statement

The authors declare that the research was conducted in the absence of any commercial or financial relationships that could be construed as a potential conflict of interest.

## References

[B1] Amrhein P. C. McDaniel M. A. Waddill P. (2002). Revisiting the picture-superiority effect in symbolic comparisons: do pictures provide privileged access? J. Exp. Psychol. Learn. Mem. Cogn. 28, 843–857 10.1037/0278-7393.28.5.843 12219794

[B2] Baddeley A. D. Emslie H. Nimmo-Smith I. (1994). Doors and People: A test of Visual and Verbal Recall and Recognition. Bury St. Edmunds: Thames Valley Test Company

[B3] Binder J. R. Desai R. H. Graves W. W. Conant L. L. (2009). Where is the semantic system? A critical review and meta-analysis of 120 functional neuroimaging studies. Cereb. Cortex 19, 2767–2796 10.1093/cercor/bhp055 19329570 PMC2774390

[B4] Booth J. R. Burman D. D. Meyer J. R. Gitelman D. R. Parrish T. B. Mesulam M. M. (2002). Functional anatomy of intra- and cross-modal lexical tasks. Neuroimage 16, 7–22 10.1006/nimg.2002.1081 11969313

[B5] Booth J. R. Burman D. D. Meyer J. R. Lei Z. Choy J. Gitelman D. R. (2003). Modality-specific and -independent developmental differences in the neural substrate for lexical processing. J. Neurolinguistics 16, 383–405 10.1016/S0911-6044(03)00019-8

[B6] Borowsky R. Esopenko C. Cummine J. Sarty G. E. (2007). Neural representations of visual words and objects: a functional MRI study on the modularity of reading and object processing. Brain Topogr. 20, 89–96 10.1007/s10548-007-0034-1 17929158

[B7] Bright P. Moss H. Tyler L. K. (2004). Unitary vs multiple semantics: PET studies of word and picture processing. Brain Lang. 89, 417–432 10.1016/j.bandl.2004.01.010 15120534

[B8] Brownsett S. L. Wise R. J. (2010). The contribution of the parietal lobes to speaking and writing. Cereb. Cortex 20, 517–523 10.1093/cercor/bhp120 19531538 PMC2820696

[B9] Buchel C. Price C. Friston K. (1998). A multimodal language region in the ventral visual pathway. Nature 394, 274–277 10.1038/28389 9685156

[B10] Cabeza R. Ciaramelli E. Moscovitch M. (2012). Cognitive contributions of the ventral parietal cortex: an integrative theoretical account. Trends Cogn. Sci. 16, 338–352 10.1016/j.tics.2012.04.008 22609315 PMC3367024

[B11] Caramazza A. (1996). Neuropsychology. Pictures, words and the brain. Nature 383, 216–217 10.1038/383216a0 8805691

[B12] Caramazza A. Hillis A. E. (1990). Where do semantic errors come from? Cortex 26, 95–122 10.1016/S0010-9452(13)80077-9 2354648

[B13] Caramazza A. Hillis A. E. Rapp B. C. Romani C. (1990). The multiple semantics hypothesis: multiple confusions? Cogn. Neuropsychol. 7, 161–189 10.1080/02643299008253441

[B14] Caramazza A. Mahon B. Z. (2006). The organisation of conceptual knowledge in the brain: the future's past and some future directions. Cogn. Neuropsychol. 23, 13–38 10.1080/02643290542000021 21049320

[B15] Chao L. L. Haxby J. V. Martin A. (1999). Attribute-based neural substrates in temporal cortex for perceiving and knowing about objects. Nat. Neurosci. 2, 913–919 10.1038/13217 10491613

[B16] Chao L. L. Martin A. (2000). Representation of manipulable man-made objects in the dorsal stream. Neuroimage 12, 478–484 10.1006/nimg.2000.0635 10988041

[B17] Chauncey K. Holcomb P. J. Grainger J. (2009). Primed picture naming within and across languages: an ERP investigation. Cogn. Affect. Behav. Neurosci. 9, 286–303 10.3758/CABN.9.3.286 19679764

[B18] Chou T. L. Chen C. W. Wu M. Y. Booth J. R. (2009). The role of inferior frontal gyrus and inferior parietal lobule in semantic processing of Chinese characters. Exp. Brain Res. 198, 465–475 10.1007/s00221-009-1942-y 19618170 PMC3277261

[B19] Coppens P. Frisinger D. (2005). Category-specific naming effect in non-brain-damaged individuals. Brain Lang. 94, 61–71 10.1016/j.bandl.2004.11.008 15896384

[B20] Cox R. W. (1996). AFNI: software for analysis and visualization of functional magnetic resonance neuroimages. Comput. Biomed. Res. 29, 162–173 10.1006/cbmr.1996.0014 8812068

[B21] Curran T. Dien J. (2003). Differentiating amodal familiarity from modality-specific memory processes: an ERP study. Psychophysiology 40, 979–988 10.1111/1469-8986.00116 14986851 PMC1413574

[B22] Daltrozzo J. Claude L. Tillmann B. Bastuji H. Perrin F. (2012). Working memory is partially preserved during sleep. PLoS ONE 7:e50997 10.1371/journal.pone.0050997 23236418 PMC3517624

[B23] Danker J. F. Hwang G. M. Gauthier L. Geller A. Kahana M. J. Sekuler R. (2008). Characterizing the ERP Old-New effect in a short-term memory task. Psychophysiology 45, 784–793 10.1111/j.1469-8986.2008.00672.x 18513360 PMC2828935

[B24] Davis M. H. Meunier F. Marslen-Wilson W. D. (2004). Neural responses to morphological, syntactic, and semantic properties of single words: an fMRI study. Brain Lang. 89, 439–449 10.1016/S0093-934X(03)00471-1 15120536

[B25] Dilkina K. Lambon Ralph M. A. (2012). Conceptual structure within and between modalities. Front. Hum. Neurosci. 6:333. 10.3389/fnhum.2012.00333 23293593 PMC3534132

[B26] Duzel E. Cabeza R. Picton T. W. Yonelinas A. P. Scheich H. Heinze H. J. (1999). Task-related and item-related brain processes of memory retrieval. Proc. Natl. Acad. Sci. U.S.A. 96, 1794–1799 10.1073/pnas.96.4.1794 9990104 PMC15598

[B27] Filliter J. H. McMullen P. A. Westwood D. (2005). Manipulability and living/non-living category effects on object identification. Brain Cogn. 57, 61–65 10.1016/j.bandc.2004.08.022 15629216

[B28] Friederici A. D. (2002). Towards a neural basis of auditory sentence processing. Trends Cogn. Sci. 6, 78–84 10.1016/S1364-6613(00)01839-8 15866191

[B29] Fuggetta G. Rizzo S. Pobric G. Lavidor M. Walsh V. (2009). Functional representation of living and nonliving domains across the cerebral hemispheres: a combined event-related potential/transcranial magnetic stimulation study. J. Cogn. Neurosci. 21, 403–414 10.1162/jocn.2008.21030 18510439

[B30] Gardner H. E. Lambon Ralph M. A. Dodds N. Jones T. Ehsan S. Jefferies E. (2012). The differential contributions of pFC and temporo-parietal cortex to multimodal semantic control: exploring refractory effects in semantic aphasia. J. Cogn. Neurosci. 24, 778–793 10.1162/jocn_a_00184 22220727

[B31] Hajcak G. Dunning J. P. Foti D. (2009). Motivated and controlled attention to emotion: time-course of the late positive potential. Clin. Neurophysiol. 120, 505–510 10.1016/j.clinph.2008.11.028 19157974

[B32] Heekeren H. R. Marrett S. Ruff D. A. Bandettini P. A. Ungerleider L. G. (2006). Involvement of human left dorsolateral prefrontal cortex in perceptual decision making is independent of response modality. Proc. Natl. Acad. Sci. U.S.A. 103, 10023–10028 10.1073/pnas.0603949103 16785427 PMC1479865

[B33] Hickok G. Poeppel D. (2007). The cortical organization of speech processing. Nat. Rev. Neurosci. 8, 393–402 10.1038/nrn2113 17431404

[B34] Hocking J. Price C. J. (2009). Dissociating verbal and nonverbal audiovisual object processing. Brain Lang. 108, 89–96 10.1016/j.bandl.2008.10.005 19101025 PMC2693664

[B35] Holcomb P. J. Kounios J. Anderson J. E. West W. C. (1999). Dual-coding, context-availability, and concreteness effects in sentence comprehension: an electrophysiological investigation. J. Exp. Psychol. Learn. Mem. Cogn. 25, 721–742 10.1037/0278-7393.25.3.721 10368929

[B36] Holcomb P. J. Neville H. J. (1990). Auditory and visual semantic priming in lexical decision: a comparison using event-related brain potentials. Lang. Cogn. Process. 5, 281–312 10.1080/0169096900840706518056222

[B37] Huth A. G. Nishimoto S. Vu A. T. Gallant J. L. (2012). A continuous semantic space describes the representation of thousands of object and action categories across the human brain. Neuron 76, 1210–1224 10.1016/j.neuron.2012.10.014 23259955 PMC3556488

[B38] Jolicoeur P. Gluck M. A. Kosslyn S. M. (1984). Pictures and names: making the connection. Cogn. Psychol. 16, 243–275 10.1016/0010-0285(84)90009-4 6734136

[B39] Kaplan E. Goodglass H. Weintraub S. (1983). Boston Naming Test. Philadelphia, PA: Lea and Febiger

[B40] Kayser J. Fong R. Tenke C. E. Bruder G. E. (2003). Event-related brain potentials during auditory and visual word recognition memory tasks. Brain Res. Cogn. Brain Res. 16, 11–25 10.1016/S0926-6410(02)00205-7 12589884

[B41] Kiefer M. (2001). Perceptual and semantic sources of category-specific effects: event-related potentials during picture and word categorization. Mem. Cognit. 29, 100–116 10.3758/BF03195745 11277454

[B42] Kiefer M. (2005). Repetition-priming modulates category-related effects on event-related potentials: further evidence for multiple cortical semantic systems. J. Cogn. Neurosci. 17, 199–211 10.1162/0898929053124938 15811233

[B43] Kissler J. Herbert C. Winkler I. Junghofer M. (2009). Emotion and attention in visual word processing: an ERP study. Biol. Psychol. 80, 75–83 10.1016/j.biopsycho.2008.03.004 18439739

[B44] Kos M. Van Den Brink D. Hagoort P. (2012). Individual variation in the late positive complex to semantic anomalies. Front. Psychol. 3:318. 10.3389/fpsyg.2012.00318 22973249 PMC3434872

[B45] Kounios J. Holcomb P. J. (1994). Concreteness effects in semantic processing: ERP evidence supporting dual-coding theory. J. Exp. Psychol. Learn. Mem. Cogn. 20, 804–823 10.1037/0278-7393.20.4.804 8064248

[B46] Kutas M. Federmeier K. D. (2011). Thirty years and counting: finding meaning in the N400 component of the event-related brain potential (ERP). Annu. Rev. Psychol. 62, 621–647 10.1146/annurev.psych.093008.131123 20809790 PMC4052444

[B47] Leonard M. K. Ferjan Ramirez N. Torres C. Travis K. E. Hatrak M. Mayberry R. I. (2012). Signed words in the congenitally deaf evoke typical late lexicosemantic responses with no early visual responses in left superior temporal cortex. J. Neurosci. 32, 9700–9705 10.1523/JNEUROSCI.1002-12.2012 22787055 PMC3418348

[B48] Leonard M. K. Torres C. Travis K. E. Brown T. T. Hagler D. J. Jr. Dale A. M. (2011). Language proficiency modulates the recruitment of non-classical language areas in bilinguals. PLoS ONE 6:e18240 10.1371/journal.pone.0018240 21455315 PMC3063800

[B49] Marinkovic K. Dhond R. P. Dale A. M. Glessner M. Carr V. Halgren E. (2003). Spatiotemporal dynamics of modality-specific and supramodal word processing. Neuron 38, 487–497 10.1016/S0896-6273(03)00197-1 12741994 PMC3746792

[B50] Markmen E. (1991). Basic, Superordinate, and Subordinate Level Categorization. Cambridge: The MIT Press

[B51] Martin A. (2007). The representation of object concepts in the brain. Annu. Rev. Psychol. 58, 25–45 10.1146/annurev.psych.57.102904.190143 16968210

[B52] Martin A. Chao L. L. (2001). Semantic memory and the brain: structure and processes. Curr. Opin. Neurobiol. 11, 194–201 10.1016/S0959-4388(00)00196-3 11301239

[B53] Mehta J. Jerger S. Jerger J. Martin J. (2009). Electrophysiological correlates of word comprehension: event-related potential (ERP) and independent component analysis (ICA). Int. J. Audiol. 48, 1–11 10.1080/14992020802527258 19173108

[B54] Mervis C. Crisafi M. (1982). Order of acquisition of subordinate-, basic- and superordinate-level categories. Child Dev. 53, 258–266 10.2307/1129660

[B55] Moore C. J. Price C. J. (1999). Three distinct ventral occipitotemporal regions for reading and object naming. Neuroimage 10, 181–192 10.1006/nimg.1999.0450 10417250

[B56] Oldfield R. C. (1971). The assessment and analysis of handedness: the Edinburgh inventory. Neuropsychologia 9, 97–113 10.1016/0028-3932(71)90067-4 5146491

[B57] Olichney J. M. Van Petten C. Paller K. A. Salmon D. P. Iragui V. J. Kutas M. (2000). Word repetition in amnesia. Electrophysiological measures of impaired and spared memory. Brain 123(Pt 9), 1948–1963 10.1093/brain/123.9.1948 10960058

[B58] Paivio A. (1971). Imagery and Verbal Processes. New York, NY: Holt, Rinehart and Winston

[B59] Paivio A. (1986). Mental Representations: A Dual-Coding Approach. New York, NY: Oxford University Press

[B60] Paivio A. (2010). Dual coding theory and the mental lexicon. Ment. Lexicon 5, 205–230 10.1075/ml.5.2.04pai21428998

[B61] Paivio A. Yuille J. C. Madigan S. A. (1968). Concreteness, imagery, and meaningfulness values for 925 nouns. J. Exp. Psychol. 76(Suppl.), 1–25 10.1037/h0025327 5672258

[B62] Patterson K. Nestor P. J. Rogers T. T. (2007). Where do you know what you know? The representation of semantic knowledge in the human brain. Nat. Rev. Neurosci. 8, 976–987 10.1038/nrn2277 18026167

[B63] Postler J. De Bleser R. Cholewa J. Glauche V. Hamzei F. Weiller C. (2003). Neuroimaging the semantic system(s). Aphasiology 17, 799–814 10.1080/02687030344000265

[B64] Randolph C. Tierney M. C. Mohr E. Chase T. N. (1998). The Repeatable Battery for the Assessment of Neuropsychological Status (RBANS): preliminary clinical validity. J. Clin. Exp. Neuropsychol. 20, 310–319 10.1076/jcen.20.3.310.823 9845158

[B65] Rogers T. T. Patterson K. (2007). Object categorization: reversals and explanations of the basic-level advantage. J. Exp. Psychol. Gen. 136, 451–469 10.1037/0096-3445.136.3.451 17696693

[B66] Rorden C. Brett M. (2000). Stereotaxic display of brain lesions. Behav. Neurol. 12, 191–200 11568431 10.1155/2000/421719

[B67] Sartori G. Job R. Miozzo M. Zago S. Marchiori G. (1993). Category-specific form-knowledge deficit in a patient with herpes simplex virus encephalitis. J. Clin. Exp. Neuropsychol. 15, 280–299 10.1080/01688639308402563 8491851

[B68] Schmitt B. M. Munte T. F. Kutas M. (2000). Electrophysiological estimates of the time course of semantic and phonological encoding during implicit picture naming. Psychophysiology 37, 473–484 10.1111/1469-8986.3740473 10934906

[B69] Seghier M. L. (2013). The angular gyrus: multiple functions and multiple subdivisions. Neuroscientist 19, 43–61 10.1177/1073858412440596 22547530 PMC4107834

[B70] Sevostianov A. Horwitz B. Nechaev V. Williams R. Fromm S. Braun A. R. (2002). fMRI study comparing names versus pictures of objects. Hum. Brain. Mapp. 16, 168–175 10.1002/hbm.10037 12112770 PMC6871815

[B71] Snodgrass J. G. Vanderwart M. (1980). A standardized set of 260 pictures: norms for name agreement, image agreement, familiarity, and visual complexity. J. Exp. Psychol. Hum. Learn. 6, 174–215 10.1037/0278-7393.6.2.174 7373248

[B72] Stewart F. Parkin A. J. Hunkin N. M. (1992). Naming impairments following recovery from herpes simplex encephalitis: category-specific? Q. J. Exp. Psychol. A 44, 261–284 10.1080/02724989243000037 1565801

[B73] Tanaka J. Luu P. Weisbrod M. Kiefer M. (1999). Tracking the time course of object categorization using event-related potentials. Neuroreport 10, 829–835 10.1097/00001756-199903170-00030 10208556

[B74] Thierry G. Price C. J. (2006). Dissociating verbal and nonverbal conceptual processing in the human brain. J. Cogn. Neurosci. 18, 1018–1028 10.1162/jocn.2006.18.6.1018 16839307

[B75] Tyler L. K. Stamatakis E. A. Dick E. Bright P. Fletcher P. Moss H. (2003). Objects and their actions: evidence for a neurally distributed semantic system. Neuroimage 18, 542–557 10.1016/S1053-8119(02)00047-2 12595206

[B76] Uddin L. Q. Supekar K. Amin H. Rykhlevskaia E. Nguyen D. A. Greicius M. D. (2010). Dissociable connectivity within human angular gyrus and intraparietal sulcus: evidence from functional and structural connectivity. Cereb. Cortex 20, 2636–2646 10.1093/cercor/bhq011 20154013 PMC2951845

[B77] Vandenberghe R. Price C. Wise R. Josephs O. Frackowiak R. S. (1996). Functional anatomy of a common semantic system for words and pictures. Nature 383, 254–256 10.1038/383254a0 8805700

[B78] Van Strien J. W. Verkoeijen P. P. Van Der Meer N. Franken I. H. (2007). Electrophysiological correlates of word repetition spacing: ERP and induced band power old/new effects with massed and spaced repetitions. Int. J. Psychophysiol. 66, 205–214 10.1016/j.ijpsycho.2007.07.003 17688964

[B79] Vigneau M. Beaucousin V. Herve P. Y. Duffau H. Crivello F. Houde O. (2006). Meta-analyzing left hemisphere language areas: phonology, semantics, and sentence processing. Neuroimage 30, 1414–1432 10.1016/j.neuroimage.2005.11.002 16413796

[B80] Visser M. Lambon Ralph M. A. (2011). Differential contributions of bilateral ventral anterior temporal lobe and left anterior superior temporal gyrus to semantic processes. J. Cogn. Neurosci. 23, 3121–3131 10.1162/jocn_a_00007 21391767

[B81] Von Stein A. Rappelsberger P. Sarnthein J. Petsche H. (1999). Synchronization between temporal and parietal cortex during multimodal object processing in man. Cereb. Cortex 9, 137–150 10.1093/cercor/9.2.137 10220226

[B82] Wu X. Lu J. Chen K. Long Z. Wang X. Shu H. (2009). Multiple neural networks supporting a semantic task: an fMRI study using independent component analysis. Neuroimage 45, 1347–1358 10.1016/j.neuroimage.2008.12.050 19166946

