# Supplementary material for: Spatial and temporal features of superordinate semantic processing studied with fMRI and EEG
Source: Front Hum Neurosci. 2013 Jul 1;7:293. doi: 10.3389/fnhum.2013.00293 (PMC3696724; doi:10.3389/fnhum.2013.00293)
Supplement: Supplementary file 1 [file DataSheet1.DOCX]

Supplementary Materials

**fMRI responses unique to auditory words and visual pictures**

Responses that were significantly greater than baseline (p<0.01, corrected) for either visual pictures or auditory words, but not for both, were observed. For auditory words, the largest and most robust activations were found in the left superior and middle temporal gyri. The most robust activations that unique for visual pictures were found in the right fusiform and inferior occipital gyri bilaterally.

**EEG/ERP responses unique to auditory words and visual pictures**

Responses that were significantly larger than baseline for either visual pictures or auditory words, but not both, were also observed. Visual pictures (vs. pseudo-stimuli) evoked a negative deflection in both left and right occipital channels, maximal in POz, beginning at 200 ms and peaking at 310 ms following stimulus onset. Mean amplitudes were compared, and showed a significant main effect of condition, real images eliciting a significantly greater negative deflection than pseudo images [F(1,65) = 21.86 p<0.0001]. No interaction was observed between condition and channel.

Auditory words (vs. pseudo-stimuli) evoked a negative deflection in left temporal channels, maximal at T7, beginning at 400 ms and peaking at 490 ms after stimulus onset. Comparison of mean amplitudes showed a significant main effect for condition, with words eliciting a greater negative amplitude than pseudo stimuli [F(1,13)=6.18,p=0.0273]. No interaction was observed between condition and channel.
